# Statin use and the risk of acute kidney injury in older adults

**DOI:** 10.1186/s12882-019-1280-7

**Published:** 2019-03-25

**Authors:** Marcello Tonelli, Anita M. Lloyd, Aminu K. Bello, Matthew T. James, Scott W. Klarenbach, Finlay A. McAlister, Braden J. Manns, Ross T. Tsuyuki, Brenda R. Hemmelgarn, for the Alberta Kidney Disease Network

**Affiliations:** 1 Department of Medicine, University of Calgary, 7th Floor, TRW Building, 3280 Hospital Drive NW, Calgary, Alberta T2N 4Z6 Canada; 2 Department of Medicine, University of Alberta, Edmonton, Canada

**Keywords:** Statins, Kidney injury-acute, Older adults

## Abstract

**Background:**

As more patients at lower cardiovascular (CV) risk are treated with statins, the balance between cardiovascular benefits and the risk of adverse events becomes increasingly important.

**Methods:**

We did a population-based cohort study (May 1, 2002 to March 30, 2013) using province-wide laboratory and administrative data in Alberta. We studied new statin users aged 66 years of age and older who were not receiving renal replacement therapy at baseline. We assessed statin use at 30-day intervals to allow time-varying assessment of statin exposure in Cox proportional hazards models that examined the relation between statin use and hospitalization with acute kidney injury (AKI).

**Results:**

Of the 128,140 new statin users, 47 and 46% were prescribed high- and medium-intensity regimens at the index date. During median follow-up of 4.6 years (interquartile range 2.2, 7.4), 9118 individuals were hospitalized for AKI. Compared to non-use, the use of high- and medium-intensity statin regimens was associated with significant increases in the adjusted risks of hospitalization with AKI: hazard ratios 1.16 [95% confidence interval (CI) 1.10, 1.23] and 1.07 (95% CI 1.01, 1.13), respectively. Risks of AKI were higher among women than men, and among users of angiotensin converting enzyme inhibitors/angiotensin receptor blockers than non-users, and among diuretic users (p for interaction 0.002, 0.01, and 0.04 respectively).

**Conclusions:**

We found a graded, independent association between the intensity of statin use and the risk of hospitalization with AKI, although the absolute magnitude of the excess risk was small.

**Electronic supplementary material:**

The online version of this article (10.1186/s12882-019-1280-7) contains supplementary material, which is available to authorized users.

## Background

Randomized controlled trials (RCTs) demonstrate that statins reduce cardiovascular (CV) risk in diverse clinical populations – including reductions in all-cause mortality, myocardial infarction, stroke, and the need for coronary revascularization [1]. Although statins are undoubtedly beneficial for people at risk of CV events, they also have side effects that may partially offset their benefits [2, 3]. As the prevalence of statin use increases among patients at lower CV risk [4], the risk of adverse events becomes increasingly important for accurately assessing net clinical benefit.

Acute kidney injury (AKI) is associated with adverse clinical outcomes and high healthcare costs [5, 6]. Even mild forms of AKI (increases in serum creatinine of 26 μmol/L) are associated with excess mortality, prolonged length of hospital stay, and high healthcare costs [7]. Earlier studies done with less potent statin regimens (pravastatin 40 mg daily or equivalent) suggested that statin treatment might decrease the risk of AKI in people at high CV risk [8]. More recent studies in which more intensive statin treatment were more frequently used suggest that statins actually increase the risk of AKI [9, 10]. However, factors that influence any excess risk associated with statin use have not been conclusively determined.

We used a unique population-based dataset with data on clinical information, medication use, laboratory results and health outcomes to test the hypothesis that the risk of AKI due to statin treatment is regimen-specific, with the highest risk observed for higher doses and for high potency statins. An important secondary hypothesis was that any apparent excess risk of AKI associated with statin use was confounded by concomitant use of angiotensin converting enzyme inhibitors (ACEI), angiotensin receptor blockers (ARB) or diuretics.

## Methods

### Alberta Kidney Disease Network (AKDN) database

We used a previously described population-based database [11], which incorporates data from Alberta Health (AH; the provincial health ministry) such as demographics, physician claims, hospitalizations, ambulatory care utilization and Alberta Blue Cross Coverage for Seniors drug data; the Northern and Southern Alberta Renal Programs and the clinical laboratories in Alberta. All people registered with the Alberta Health Care Insurance Plan (AHCIP) were included in the database; all Alberta residents are eligible for the AHCIP and > 99% participate in coverage. For eligible individuals aged 65 years and older, prescription drugs dispensed from community pharmacies are adjudicated through Alberta Blue Cross. There are no premiums for this coverage and the individual shares the cost of the prescription with AH by paying 30% to a maximum of $25 for each prescription dispensed. We used the database to assemble a cohort of individuals 66 years of age and older with an incident statin prescription between 2002 and 2013 who were not previously receiving renal replacement therapy nor had a kidney transplant. We excluded people insured by Health Canada’s, First Nations and Inuit Health, Non-Insured Health Benefit Program rather than AH due to lack of drug data.

Participants entered the cohort on the date of their first statin prescription (i.e., index date) on or after their 66th birthday between May 1, 2002 and March 30, 2013. Prescriptions for any of atorvastatin, fluvastatin, lovastatin, pravastatin, rosuvastatin, or simvastatin were considered. By limiting entry to occur at least one year after eligibility for Alberta Blue Cross Coverage for Seniors commenced, we could properly assess previous statin use, using a one-year washout period. We excluded individuals who had ≥1 statin prescription during the one year period prior to their first statin prescription; we considered the remaining participants as incident statin users and included them in the main analyses (Additional file 1: Figure S1).

### Assessment of exposure to statins

We deemed exposure to statins to have commenced on the date of the first statin prescription and reassessed exposure every 30 days of follow-up. We considered individuals as statin users for the entire 30-day period if they were dispensed at least one statin tablet. Incident statin users were grouped according to the intensity of statin [12] they were receiving and could move back and forth between statin use and non-use as well as between different types of statins. The non-use periods of the time-varying statin exposure variable formed the reference group in analyses. We classified statin intensity as high, medium or low according to the National Institute for Health and Clinical Excellence (NICE) clinical guidelines on lipid modification [12] (Additional file 1: Table S1). If a non-use period was ≤90 days and was followed by a period of statin use, we assumed that these individuals were statin users during this “non-use” period. We assumed individuals were adherent with statin prescriptions if they continued to have prescriptions for statins dispensed. If in a 30-day period an individual was dispensed more than one type of statin, we classified that individual as receiving only one agent for the entire period (the agent which accounted for the majority of tablets during that period). Usage was based on dispensing events and number of days supplied (Additional file 1: Figure S2).

### Definitions

We used the Chronic Kidney Disease Epidemiology Collaboration (CKD-EPI) equation [13] and a standardized serum creatinine assay to estimate the baseline estimated glomerular filtration rate (eGFR) for each participant. The most recent outpatient serum creatinine on or within 1 year prior to the index date was selected if available. We assessed albuminuria using the most recent outpatient measurement (or the median of all multiple measurements available) on or within 1 year prior to the index date and categorized it as: normal [albumin:creatinine ratio (ACR) < 3 mg/mmol, protein:creatinine ratio (PCR) < 15 mg/mmol or urine dipstick negative], moderately increased (ACR 3–30 mg/mmol, PCR 15–50 mg/mmol or urine dipstick trace or 1+), severely increased (ACR > 30 mg/mmol, PCR > 50 mg/mmol or urine dipstick ≥2+), or not measured/available. We used ACR as the primary measure of albuminuria; if ACR was unavailable we used PCR; if PCR was unavailable we used dipstick urinalysis. We used validated algorithms based on claims and hospitalization data to classify participants regarding the baseline presence of 30 comorbidities [14]. We also classified individuals with respect to any use of ACEI, ARB or loop diuretics (oral furosemide or ethacrynic acid; as captured through dispensing events) within 1 year of the index date.

### Ascertainment of outcomes

We followed participants from their index date until the outcome of interest, date of death, initiation of chronic renal replacement, outmigration from the province or the study end (March 31, 2013). The primary study outcome was hospitalization with acute kidney injury (AKI) based on International Classification of Diseases (ICD9 or ICD10) codes obtained from Alberta Health data [15] (Additional file 1: Table S2).

### Statistical analyses

We reported baseline descriptive statistics as percentages or medians and interquartile ranges (IQR) as appropriate. We used Cox proportional hazards models to assess the relationship between statin intensity and the primary outcome of interest. We treated statin use categorized by intensity as a time-varying exposure. Covariates (eGFR, albuminuria, comorbidities, medication use) were also time-varying; they were updated for each 30-day interval and defined in the same manner as at baseline. We tested the proportional hazards assumption by assessing log-log plots.

To test our secondary hypothesis, we examined whether the relationship between statin exposure and hospitalization with AKI was modified by a) ACEI/ARB use at baseline; or b) loop diuretic use at baseline. We also examined other potential dichotomous effect modifiers as follows: c) age 66–75 years versus age > 75 years; d) sex; e) diabetic status at index; f) chronic kidney disease (CKD) status at index (where individuals were classified as having CKD if either their baseline eGFR was less than 60 ml/min/1. 73m^2^ or they had “moderately increased” or worse albuminuria); and g) albuminuria status at baseline (where individuals had albuminuria if they had “moderately increased” or worse albuminuria). Finally, to test whether AKI apparently due to statin use was actually due to cardiovascular events or interventions that were associated with both initiation of statins and hospitalization with AKI, we stratified on the presence of i) cardiovascular events. Cardiovascular events of interest included cerebrovascular vascular accident (CVA), myocardial infarction (MI), coronary artery bypass grafting (CABG), percutaneous coronary intervention (PCI), or coronary angiogram. The presence or absence of these cardiovascular events was classified during the entire follow-up period. Analyses a) to i) were done by including an interaction term between the stratification variable and statin exposure in each model.

We did several sensitivity analyses, including a) repeating the primary analyses in individuals without previous cardiovascular disease as of the index date; and b) assessing the relation between statin intensity and hospitalization for AKI requiring dialysis (based on ICD 9 or ICD 10 codes in Additional file 1: Table S2); and c) repeating the primary analyses based on defining statin exposure in terms of medication possession ratio [16]. We defined cardiovascular disease as any of: acute myocardial infarction, percutaneous coronary intervention, coronary artery bypass grafting, coronary catheterization, heart failure or stroke/transient ischemic attack. We assessed medication possession ratio (MPR) as a percentage at each 30-day interval and was defined as the “number of tablets” divided by “days in period”. We categorized MPR into three categories as follows: MPR ≥ 75%, MPR 1 to 75%, and non-use (where as before, these were the periods where no tablets were taken). If a non-use period was ≤90 days and was followed by a period of statin use, the last available MPR value was carried forward into this period.

We also compared the characteristics of statin users to an age- and sex-matched cohort of randomly selected non-users. First, random index dates were generated for the non-users according to the distribution of index dates of the statin users; the statin users were each matched to a non-user (if a match was available) based on age (in 5 year increments) and sex. Characteristics between statin users and non-users were compared using standardized differences [17].

We did statistical analyses using Stata 13.1 MP software (www.stata.com). The institutional review boards at the Universities of Calgary and Alberta approved the study (REB14–0884, Pro00053469) with a waiver of consent granted.

## Results

There were 128,140 incident statin users; the characteristics of statin users are compared to an age- and sex- matched cohort of randomly selected non-users is presented in Additional file 1: Table S3. As expected, non-users were significantly less likely than users to have prior vascular disease. Baseline characteristics of the statin users that were the focus of analysis are presented according to the intensity of statin use (which reflects potency and dose) at the index date (Table 1). The statin regimens were 47% high-intensity and 46% medium-intensity; < 7% of individuals were initially prescribed a low-intensity regimen. Individuals taking a high-intensity regimen were similar in age and diabetic status but there were more males in comparison to those taking medium- or low-intensity regimens. The former generally had similar levels of baseline comorbidities in comparison to the latter groups except for acute myocardial infarction and chronic kidney disease which were all more common in the high intensity group. During a median follow-up of 4.6 years (IQR 2.2, 7.4), total follow-up time approximately 624,000 person-years, 9118 individuals were hospitalized for AKI. The rates of hospitalization for AKI (per 1000 person years) were 17.1 [95% confidence interval (CI) 16.6, 17.7] for high-intensity statin use, 13.8 (95% CI 13.3, 14.4) for medium-intensity statin use, 13.3 (95% CI 12.1, 14.7) for low-intensity statin use, and 12.7 (95% CI 12.2, 13.2) for no statin use.

**Table 1:** Baseline characteristics at index date

	High-intensity	Medium-intensity	Low-intensity
	*n* = 60,625	*n* = 58,919	*n* = 8596
Age, years^a^	72.7 (68.6, 78.1)	73.1 (69, 78.5)	73.4 (68.9, 78.8)
Female	45	53	55
Hypertension	73	71	70
Diabetes	27	29	27
Statin			
Rosuvastatin	≥10 mg: 44	< 10 mg: 19	0
Atorvastatin	≥20 mg: 55	< 20 mg: 62	0
Simvastatin	≥80 mg: 0.3	20–40 mg: 20	< 20 mg: 40
Lovastatin	0	40 mg: 0.2	< 40 mg: 4
Pravastatin	0	0	≤40 mg: 52
Fluvastatin	0	80 mg: 0.1	≤40 mg: 4
Comorbidities			
Alcohol misuse	2	2	2
Asthma	4	3	3
Atrial fibrillation	12	10	10
Cancer, lymphoma	1	1	1
Cancer, metastatic	1	1	1
Cancer, non-metastatic	7	6	6
Chronic heart failure	14	11	12
Chronic kidney disease	43	39	34
Chronic pain	17	18	18
Chronic pulmonary disease	21	19	18
Chronic viral hepatitis B	0.02	0.04	0.05
Cirrhosis	0.2	0.2	0.3
Dementia	4	3	3
Depression	9	9	9
Epilepsy	1	1	1
Hypothyroidism	14	14	14
Inflammatory bowel disease	1	1	1
Irritable bowel syndrome	2	2	2
Multiple sclerosis	0.4	0.4	0.4
Myocardial infarction	17	8	7
Parkinson’s disease	1	1	2
Peptic ulcer disease	1	1	1
Peripheral vascular disease	3	3	2
Psoriasis	1	1	1
Rheumatoid Arthritis	4	4	3
Schizophrenia	1	1	1
Severe constipation	2	2	2
Stroke or TIA	18	17	16
Proteinuria			
Not measured	42	41	51
Normal	46	47	39
Moderately increased	10	10	8
Severely increased	2	2	2
Medications			
ACEI/ARB	65	58	56
Loop diuretics	13	11	11
eGFR ml/min/1. 73m^2^			
Not measured	25	27	37
< 15	0.2	0.2	0.3
15–29	2	2	2
30–44	6	6	6
45–59	15	15	13
60–89	45	44	37
≥ 90	7	6	5

Data expressed as %, except ^a^median (interquartile range). Totals do not always add to 100% because of rounding. See Additional file 1: Table S1 for statin intensity groupings

Proteinuria categories: Normal (ACR < 3 mg/mmol, PCR < 15 mg/mmol or urine dipstick negative), moderately increased (ACR 3–30 mg/mmol, PCR 15–50 mg/mmol or urine dipstick trace or 1+), severely increased (ACR > 30 mg/mmol, PCR > 50 mg/mmol or urine dipstick ≥2+)

*ACEI* angiotensin converting enzyme inhibitors, *ACR* albumin creatinine ratio, *ARB* angiotensin receptor blockers, *eGFR* estimated glomerular filtration rate, *PCR* protein:creatinine ratio, *TIA* transient ischemic attack

### Association between statin use, statin regimen intensity, statin potency, and the risk of AKI

Compared to non-use, use of any statin (all types; all doses) was associated with a significant increase in the unadjusted and adjusted risks of hospitalization with AKI [hazard ratio (HR) 1.22 (95% confidence interval CI 1.16, 1.28), and HR 1.11 (95% CI 1.06, 1.17)], respectively.

High-intensity regimens were associated with a significantly increased unadjusted risk of hospitalization with AKI [HR 1.35 (95% CI, 1.28, 1.43) vs non-use]. Similarly, the unadjusted HR for the risk of hospitalization with AKI for medium-intensity users versus non-use was also significant [HR 1.09 (95% CI 1.03, 1.16)] (Table 2). After adjustment, the adjusted hazard ratios (aHR) for the risk of hospitalization with AKI were attenuated but remained significantly increased (high-intensity versus non-use aHR 1.16 (95% CI 1.10, 1.23); medium-intensity versus non-use aHR 1.07 (95% CI 1.01, 1.13). There were no differences in the risk of hospitalization with AKI between low-intensity users and non-use (Table 2).

**Table 2:** Hazard ratios (95% CI) for the risk of hospitalization with acute kidney injury

	Unadjusted HR (95% CI)	Fully-adjusted* HR (95% CI)
High-intensity	1.35 (1.28, 1.43)	1.16 (1.10, 1.23)
Medium-intensity	1.09 (1.03, 1.16)	1.07 (1.01, 1.13)
Low-intensity	1.04 (0.93, 1.15)	1.03 (0.93, 1.15)
Non-use	1 (referent)	1 (referent)
P for trend	< 0.001	< 0.001

*CI* confidence interval, *HR* hazard ratio

*Adjusted for covariates in Table 1

### Effect modification

**Fig. 1:**
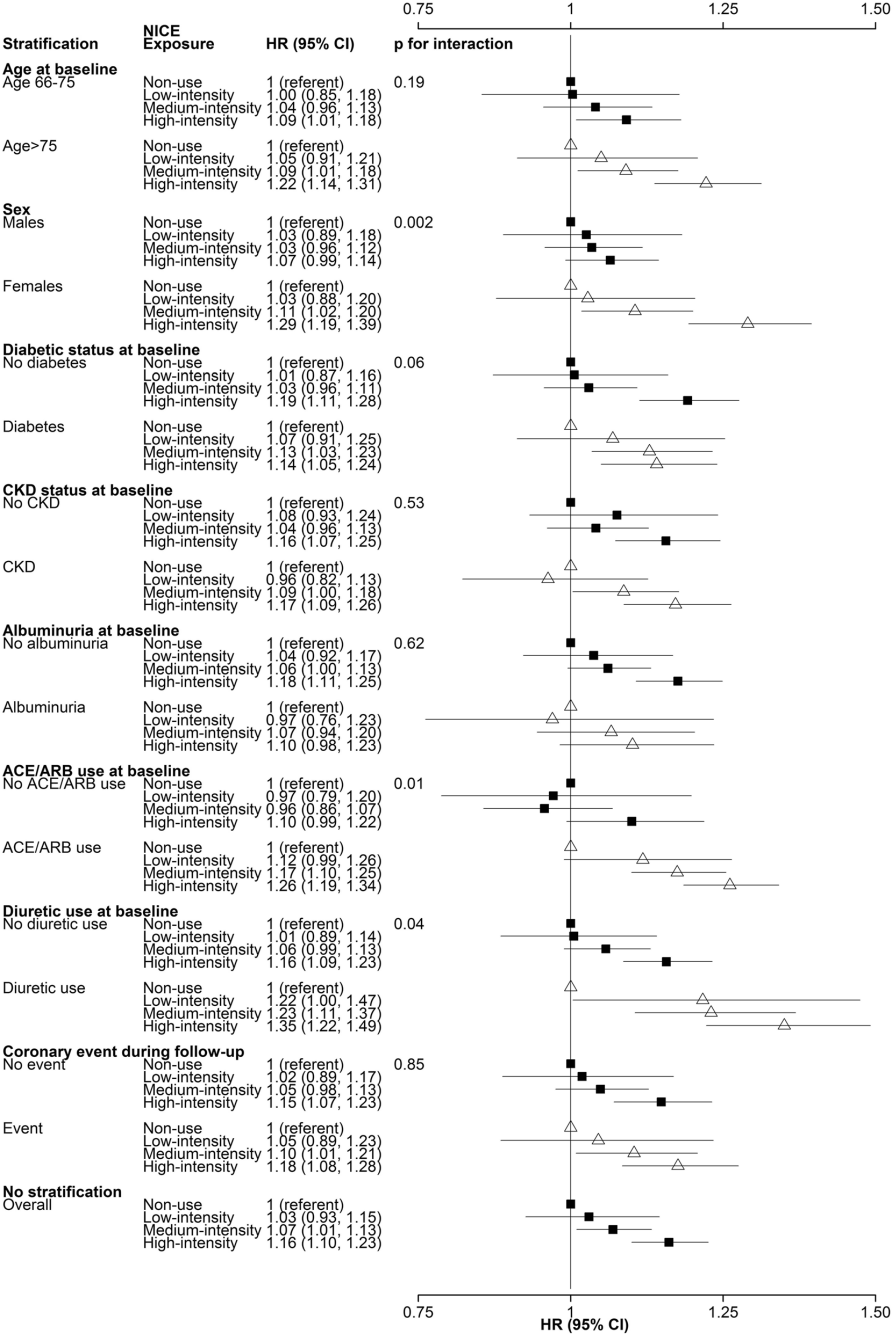
Effect modification adjusted analyses. *ACEI* angiotensin converting enzyme inhibitors, *ARB* angiotensin receptor blockers, *CI* confidence interval, *CKD* chronic kidney disease, *HR* hazard ratio, *NICE* National Institute of Clinical Excellence

In sensitivity analyses, we searched for clinical characteristics that might modify the association between statin regimen intensity and the risk of hospitalization with AKI. Of 8 characteristics considered, sex, ACEI/ARB use and loop diuretic use significantly modified these associations (Fig. 1). Specifically, the dose-adjusted risk of hospitalization with AKI associated with statin use was substantially greater in women than in men (p for interaction 0.002). While the absolute risk of hospitalization for AKI in women was smaller than in men, compared with non-use, the risk of hospitalization with AKI associated with high-intensity statin use among women was 1.29 (95% CI 1.19, 1.39) but only 1.07 (95% CI 0.99, 1.14) in men. Similarly, the increased risk of hospitalization with AKI associated with high-intensity statin use was 1.26 (95% CI 1.19, 1.34) among those using ACEI or ARB, but only 1.10 (95% CI 0.99, 1.22) among those not using the latter (p for interaction 0.01). There was also a significant interaction between diuretic use and the risk of hospitalization with AKI associated with statin use (p for interaction 0.04). Among those using a diuretic at the index date, the risk of hospitalization with AKI with high-intensity statin use was 1.35 (95% CI 1.22, 1.49) and only 1.16 (95% CI 1.09, 1.23) among those not using a diuretic.

### Other sensitivity analyses

Compared to non-use, use of any statin (all types; all doses) was associated with a significant increase in the unadjusted risk of hospitalization with AKI *requiring dialysis* but not the adjusted risk [hazard ratio (HR) 1.45 (95% confidence interval CI 1.06, 1.98), and HR 1.15 (95% CI 0.83, 1.59)], respectively. Neither low-, medium- nor high-intensity statin use were associated with adjusted risk of AKI requiring dialysis (data not shown). Compared to non-use periods, MPR of statins between 1 to 75% and ≥ 75% were both associated with a significantly increased adjusted risk of hospitalization with AKI [HR 1.23 (95% CI 1.15, 1.33)] and [HR 1.09 (95% CI 1.04, 1.15), respectively. Analyses examining the risk of hospitalization with AKI in the subgroup of individuals without cardiovascular disease at baseline were similar in findings to the main analyses that did not make this exclusion (data not shown).

## Discussion

In this population-based study of 128,140 older incident statin users treated in a single Canadian province, we found a graded, independent association between the intensity of statin treatment and the risk of hospitalization with AKI. In addition, we found that both female sex and baseline use of ACEI or ARB or loop diuretics may modify the relation between statin treatment and the risk of hospitalization with AKI.

We speculate that the observed effect modification by sex may be due to higher blood levels of statin at a given dose among women than in men, due to the generally lower body size of women. Although we adjusted for baseline use of ACEI or ARB in all analyses, the finding that the risk of statin-associated hospitalization with AKI is substantially higher in people also using ACEI or ARB suggests the possibility that the former (and not statin use per se) may have contributed to the apparently increased risk of AKI among statin users. We also found evidence that the risk of AKI associated with statin use was modified by baseline use of diuretics.

A previous large multinational observational study (*N* = 2,008,003) found an excess risk of AKI within two years of statin initiation among high potency statin users (defined by daily doses of ≥10 mg rosuvastatin, ≥20 mg atorvastatin, or ≥ 40 mg simvastatin) compared to users of statin at lower potency, but only among people without CKD at baseline [9]. In that study, claims data rather than eGFR was used to define CKD, which may have led to inaccuracies in assessing the presence or absence of pre-existing kidney disease. Also, that study did not evaluate for effect modification by characteristics such as sex or ACEI/ARB use. These differences in study design may account for the slightly different findings between that and our study. An earlier population-based cohort study in England and Wales (N = 2,004,692) that used different methods to ascertain statin exposure and clinical outcomes also found an excess risk of acute kidney injury among statin users than non-users [10]. That study also reported a higher risk among users of higher doses than users of lower doses, although potential effect modifiers such as sex and concomitant medication use were again not evaluated. A third study done in the U. S suggested that higher doses of simvastatin were associated with a higher risk of claims for AKI during follow-up [18], although there was no association between AKI and statin use as compared to non-use. Thus, on balance, available data from observational studies suggest that more intensive exposure to statins may lead to slightly increased risk of AKI, as compared to milder exposure or no exposure.

In contrast, previous RCTs have not found a significant association between statin treatment and the risk of AKI, or an excess risk of AKI for high potency vs moderate/low potency regimens [19–21]. A recent RCT examined the risk of AKI associated with a short course of atorvastatin 40–80 mg daily during the perioperative period among adult patients receiving cardiac surgery, and found no significant effect overall (HR associated with atorvastatin 1.06 (95% CI 0.78, 1.46)) or among those who were naïve to statin treatment at baseline (HR 1.61, 95% CI 0.86, 3.81)) [22]. Although they are more methodologically robust, these RCTs had much lower statistical power than the current study, and tended to study highly selected populations. Plans to pool adverse event rates across multiple large RCTs will help to offset the latter limitation [23], and may help to resolve the apparently conflicting results between RCTs and observational studies.

Our results should be placed in the context of what is known about the cardiovascular benefits of statins: higher doses of these medications clearly lead to important reductions in clinically relevant outcomes including death, stroke, and myocardial infarction [1]. While the dose-dependent increase we observed in the risk of hospitalization with AKI for statin users was statistically significant, its magnitude was clinically small: only 107 excess AKI hospitalizations (compared to non-use) would be expected in a population of 10,000 people aged > 65 years who received high intensity statin treatment for approximately 5 years, as compared with no statin use. Put differently, the number needed to harm (NNH) for one case of AKI over 5 years is approximately 93. In comparison, assuming a conservative 20% relative risk reduction, high intensity statin use in the same 10,000 people would be expected to prevent 135 myocardial infarctions during the same period, as compared with no statin use (number needed to treat, 74).

### Strengths and limitations

Our study has important strengths, including its use of a large population-based database from a setting with universal health care coverage, its use of validated algorithms for ascertaining the presence or absence of hospitalization with AKI as well as baseline comorbidity, and its rigorous analytical methods. However, our study also has several potential limitations that should be considered when interpreting results. First, like all studies using administrative data, residual confounding is possible by unmeasured characteristics including use of over-the-counter medications such as non-steroidal anti-inflammatory medications, intercurrent illness or the underlying risk of AKI among study participants. Confounding by indication is a particular concern, but the similar findings in a sensitivity limited to individuals with known cardiovascular disease at baseline should increase confidence in our findings. Second, not all individuals had baseline eGFR or albuminuria measurement available to assess CKD status. This would not have been expected to affect our key findings regarding the association between statin use and AKI, although it might have reduced statistical power to show effect modification by baseline CKD status. It is worth noting that low eGFR does not affect blood statin levels appreciably, since these medications primarily undergo hepatic metabolism rather than renal excretion [24]. Third, we studied only people older than 66 years. Although such people account for most statin users in Alberta, whether our findings apply to younger people requires confirmation. Fourth, our algorithm for identifying AKI has limitations, including a sensitivity of approximately 35%, although it is approximately 98% specific [15], as compared with a clinical gold standard. On balance, these limitations may have led to under-recognition of milder forms of AKI (perhaps reducing statistical power), although they are unlikely to have led to bias. Fifth, we were not able to identify the cause of AKI, although it is unlikely to have been due to rhabdomyolysis, given how infrequently this event occurs [1]. Sixth, like most pharmacoepidemiological studies, our data source captured dispensing of medications from pharmacies and not actual medication use. Participants may not have taken medications that were dispensed, and dates of dispensing may not correspond to actual doses taken during each 30-day interval. Finally, we studied people from a single Canadian province and our findings may not apply to other settings.

## Conclusions

In conclusion, we found a graded, independent association between the intensity of statin use and the risk of hospitalization with AKI, although the absolute magnitude of the excess risk was small. Our findings suggest that higher dose statin use could be considered as a contributing factor to AKI in older people, especially in females or those receiving ACEI, ARB or loop diuretics. This slight increase in risk should be weighed against the known and clinically relevant benefits of statins for preventing death and significant morbidity from cardiovascular disease.

## Additional files

Additional file 1: **Figure S1.** Flow Diagram. **Figure S2.** Assessment of exposure to statins. **Table S1.** Classification of statin intensity. **Table S2.** Algorithm for identifying acute kidney injury from administrative data. **Table S3.** Baseline characteristics of statin users and non-users. (DOCX 104 kb)

## Abbreviations

ACEI: Angiotensin converting enzyme inhibitors
ACR: Albumin:creatinine ratio
AH: Alberta Health
AHCIP: Alberta Health Care Insurance Plan
aHR: Adjusted hazard ratios
AKDN: Alberta Kidney Disease Network
AKI: Acute kidney injury
ARB: Angiotensin receptor blockers
CABG: Coronary artery bypass grafting
CI: Confidence interval
CKD: Chronic kidney disease
CKD-EPI: Chronic Kidney Disease Epidemiology Collaboration
CV: Cardiovascular
CVA: Cerebrovascular vascular accident
eGFR: Estimated glomerular filtration rate
HR: Hazard ratio
ICD: International Classification of Diseases
IQR: Interquartile ranges
MI: Myocardial infarction
MPR: Medication possession ratio
NICE: National Institute for Health and Clinical Excellence
NNH: Number needed to harm
PCI: Percutaneous coronary intervention
PCR: Protein:creatinine ratio
RCTs: Randomized controlled trials
