# Supplementary material for: Statin use and the risk of acute kidney injury in older adults
Source: BMC Nephrol. 2019 Mar 25;20:103. doi: 10.1186/s12882-019-1280-7 (PMC6434639; doi:10.1186/s12882-019-1280-7)
Supplement: Supplementary file 1 — Figure S1. Flow Diagram. Figure S2. Assessment of exposure to statins. Table S1. Classification of statin intensity. Table S2. Algorithm for identifying acute kidney injury from administrative data. Table S3. Baseline characteristics of statin users and non-users. (DOCX 104 kb) [file 12882_2019_1280_MOESM1_ESM.docx]

**Additional file 1**

**Statin use and the risk of acute kidney injury in older adults**

Marcello A Tonelli, MD SM MSc, (1)

Anita M Lloyd, MSc, (2)

Aminu K Bello, MBBS PhD, (2)

Matthew T James, MD PhD, (1)

Scott W Klarenbach, MD MSc, (2)

Finlay A McAlister, MD MSc, (2)

Braden J Manns, MD MSc, (1)

Ross T Tsuyuki, PharmD MSc, (1)

Brenda R Hemmelgarn, MD PhD, (1)

for the Alberta Kidney Disease Network

**Additional file 1: Figure S1. Flow diagram**


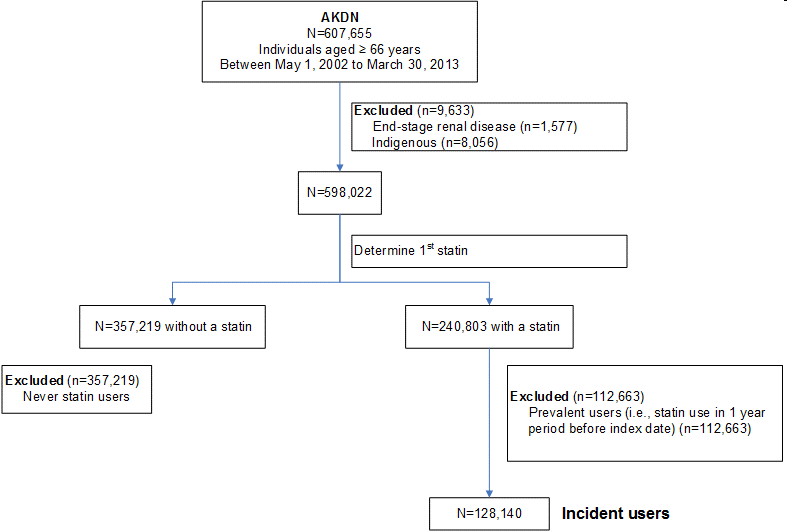


AKDN Alberta Kidney Disease Network

**Additional file 1: Figure S2. Assessment of exposure to statins**

*Individuals who had a statin prescription during the washout period were excluded – these individuals were not considered to be “incident” statin users. The washout period was defined as the 1-year period prior to the index statin prescription. Statin use and covariates were assessed at the index date and were updated every 30 days until the end of follow-up or the outcome of interest.

Qty quantity; tab tablets; simva simvastatin; atorva atorvastatin; d days

**Additional file 1: Table S1. Classification of statin intensity**

| Dose (mg) | **Rosuvastatin** | **Atorvastatin** | **Simvastatin** | **Lovastatin** | **Pravastatin** | **Fluvastatin** |
| --- | --- | --- | --- | --- | --- | --- |
| 5 |  | n/a |  | n/a | n/a | n/a |
| 10 |  |  |  | n/a |  | n/a |
| 20 |  |  |  |  |  |  |
| 40 |  |  |  |  |  |  |
| 80 | n/a |  |  | n/a | n/a |  |

**Legend:**

| n/a | Regimens not observed in AKDN participants |
| --- | --- |
|  | Low-intensity statin |
|  | Medium-intensity statin |
|  | High-intensity statin |

Classification system was adapted from National Institute of Clinical Excellence guidance[^1^](#_ENREF_1).

**Additional file 1: Table S2. Algorithm for identifying acute kidney injury from administrative data**

| **Condition** | **ICD-9** | **ICD-10** |
| --- | --- | --- |
| **Acute Kidney Injury** | Hospitalization for one of the following:  ICD-9-CM diagnosis codes: 584.5, 584.6, 584.7, 584.8, 584.9 | Hospitalization for one of the following:  ICD-10-CA diagnosis codes: N17.0, N17.1, N17.2, N17.8, N17.9 |
| **Acute Kidney Injury requiring dialysis** | Hospitalization for one of the following:  ICD-9-CM diagnosis codes: 584.5, 584.6, 584.7, 584.8, 584.9  **PLUS**  Hospitalization for one of the following:  ICD-9-CM diagnosis code: 39.95  ICD-9-CM procedure codes: V45.1, V56.0, V56.1 | Hospitalization for one of the following:  ICD-10-CA diagnosis codes: N17.0, N17.1, N17.2, N17.8, N17.9  **PLUS**  Hospitalization for one of the following:  ICD-10-CA diagnosis codes: Z99.2, Z49.0, Z49.1  CCI codes: 1.PZ.21.HQ-BR, 1.PZ.21.HQ-BS, 1.JQ.53, 1.JT.53 |

Codes may occur in any field

**Additional file 1: Table S3. Baseline characteristics of statin users and non-users**

|  | **Statin user** | **Non-user** | **Standardized difference** |
| --- | --- | --- | --- |
|  | **N=125,832** | **N=125,832** | **%** |
| Age, years* | 73.1 (69, 78.4) | 73.1 (69, 78.6) | 1.3 |
| Female | 51 | 51 | 0 |
| Hypertension | 72 | 50 | 46.1 |
| Diabetes | 28 | 11 | 44.2 |
| **Comorbidities** |  |  |  |
| Alcohol misuse | 2 | 3 | 3.0 |
| Asthma | 3 | 3 | 3.2 |
| Atrial fibrillation | 11 | 7 | 13.7 |
| Cancer, lymphoma | 1 | 1 | 0.8 |
| Cancer, metastatic | 1 | 2 | 5.3 |
| Cancer, non-metastatic | 7 | 7 | 1.4 |
| Chronic heart failure | 13 | 8 | 17.4 |
| Chronic kidney disease | 40 | 24 | 35.1 |
| Chronic pain | 17 | 14 | 10.2 |
| Chronic pulmonary disease | 20 | 17 | 7.0 |
| Chronic viral hepatitis B | 0.03 | 0.07 | 1.6 |
| Cirrhosis | 0.2 | 0.3 | 2.9 |
| Dementia | 4 | 6 | 9.6 |
| Depression | 9 | 7 | 5.7 |
| Epilepsy | 1 | 1 | 1.3 |
| Hypothyroidism | 14 | 12 | 6.7 |
| Inflammatory bowel disease | 1 | 1 | 0.4 |
| Irritable bowel syndrome | 2 | 2 | 2.0 |
| Multiple sclerosis | 0.4 | 0.6 | 3.0 |
| Myocardial infarction | 12 | 2 | 41.7 |
| Parkinson’s disease | 1 | 2 | 6.2 |
| Peptic ulcer disease | 0.5 | 0.3 | 3.0 |
| Peripheral vascular disease | 3 | 1 | 11.3 |
| Psoriasis | 1 | 1 | 1.9 |
| Rheumatoid Arthritis | 4 | 4 | 0 |
| Schizophrenia | 1 | 1 | 3.8 |
| Severe constipation | 2 | 2 | 0.6 |
| Stroke or TIA | 18 | 9 | 25.4 |
| **Proteinuria** |  |  | 56.5 |
| Not measured | 42 | 69 |  |
| Normal | 46 | 26 |  |
| Moderately increased | 10 | 4 |  |
| Severely increased | 2 | 1 |  |
| **Medications** |  |  |  |
| ACEI/ARB | 61 | 27 | 72.8 |
| Loop diuretics | 12 | 6 | 21.2 |
| **eGFR ml/min/1.73m^2^** |  |  | 68.0 |
| Not measured | 27 | 58 |  |
| <15 | 0.2 | 0.1 |  |
| 15-29 | 2 | 1 |  |
| 30-44 | 6 | 2 |  |
| 45-59 | 15 | 7 |  |
| 60-89 | 44 | 27 |  |
| ≥90 | 6 | 5 |  |

Data expressed as %, except * median (interquartile range). Totals do not always add to 100% because of rounding. Standardized differences in characteristics between statin users and non-users are shown where differences >10% suggest meaningful imbalance.

For male statin users between 66 to 70 years old, there were fewer non-users available to match on. Thus, N=2,308 statin users were not matched and excluded.

Proteinuria categories: Normal (ACR<3 mg/mmol, PCR <15 mg/mmol or urine dipstick negative), moderately increased (ACR 3-30 mg/mmol, PCR 15-50 mg/mmol or urine dipstick trace or 1+), severely increased (ACR >30 mg/mmol, PCR>50 mg/mmol or urine dipstick ≥2+)

ACEI angiotensin converting enzyme inhibitors; ACR albumin creatinine ratio; ARB angiotensin receptor blockers; eGFR estimated glomerular filtration rate; PCR protein:creatinine ratio; TIA transient ischemic attack

**References**

1. National Institute for Health and Clinical Excellence. Lipid modification: Cardiovascular risk assessment and the modification of blood lipids for the primary and secondary prevention of cardiovascular disease. 2014
